# Ten simple rules for effectively assessing lab environments

**DOI:** 10.1371/journal.pcbi.1013223

**Published:** 2025-06-24

**Authors:** Kristen M. Naegle, Jeffrey J. Saucerman, Julie Leonard-Duke, Michael Rariden, Yonathan Tamrat Aberra

**Affiliations:** Department of Biomedical Engineering, University of Virginia, Charlottesville, Virginia, United States of America; Dassault Systemes BIOVIA, UNITED STATES OF AMERICA

Pursuing a Ph.D. in biomedical sciences opens important career prospects with higher satisfaction through increased autonomy, innovation, and leadership roles [1], although it comes with economic tradeoffs during training years [2]. Once you have decided a Ph.D. is part of your career path and have a vision for what you want to gain scientifically and professionally [3–5], finding an environment well-matched to your goals is a critical step.

In an ideal world, most people should be able to thrive in most lab environments, since most lab environments should be inclusive, functional, and collaborative spaces. Such labs are a network supported by mentorship (the lab lead/principal investigator, and other lab members) that can adapt to individual needs and help people achieve their goals and aspirations. However, we also know that in practice, trainees can land in research environments that lead to unhappiness, poor mental health, and failure to thrive on their original intent [6]. This can result from several small mismatches or fewer, larger mismatches to various aspects of the lab environment (e.g., mentorship, colleagues, decision making, autonomy, or general approaches to project management and interactions) [7].

We propose that the environment has three key aspects that need to be evaluated with respect to how they will meet your needs and allow you to be successful in your pursuit of your goals—the *what*, the *how*, and the *who* of an environment. The *what* is the research area—the specific topical research field. The *how* includes your development trajectory, the specific techniques you learn, the way you work with people in and outside of your environment, and the ethics and safety considerations. The *who* includes your direct mentor(s), your colleagues, and your collaborators. We also propose that like many systems, this system can be described by Pareto optimality [8]—the best combination of factors have inherent tradeoffs where it is unlikely all three aspects of the environment will be ideal. However, if you maximize one of these at the serious expense of another aspect, then this can cause significant issues on the path to a PhD. Given the likelihood that your prior educational and other experiences have helped you better understand the *what* aspects of evaluating a lab environment, we focus here instead on the non-scientific aspects of lab environments (where a lab refers to the larger group working together under one or a few principal investigator(s)), that we think should be thoughtfully evaluated as the key step in between identifying your career and scientific goals and the process of actively pursuing those goals in graduate training [9–11].

Similarly, for PIs, the process of recruiting, training, retaining, and mentoring graduate students is also solving an optimization problem. Similar mismatches on the part of the recruit to the lab or mentorship style can cause loss of productivity and increase stress for the PI and all lab members. Hence, it is equally important for PIs to deeply evaluate the goals and culture of the lab, to seek continuous improvement in mentorship and lab management [12], but also to recognize key strengths and limitations of their mentorship and lab environments and to help identify with prospective students where these matches and mismatches might exist and how they could be strengthened throughout a training period. Herein, we have provided complementary information for PIs seeking to proactively evaluate and develop mentorship and lab cultures that directly align with their goals. To be clear, we advocate that all PIs should work on continuous improvement of mentorship and lab culture, including seeking to build diverse labs, which have shown to be more innovative and productive [13,14], while also being honest and transparent about the key aspects of decision making and values that set the overall lab direction during the process of personnel recruitment.

It is important to be honest that there are some lab environments and workplaces that would be deemed toxic by many [15–17]—these include environments where any of the following are tolerated: bullying, harassment, discrimination, abuse, unethical conduct, and unsafe working conditions. We recommend that you remove any lab with a toxic environment from your consideration – there is an unlikely chance that you will come out of that environment successfully with any of your original intent for pursuing the Ph.D. intact. All lab environments and graduate students encounter challenges from time to time, including learning to handle failure, respond to criticism, and create more successful pathways of communication. And your capacity for handling the various speedbumps in your training can be bolstered by training, such as the NIH’s Office for Intramural Training and Education (OITE) “Being a resilient scientist” [18]. But there is no place for becoming resilient to abuse or discrimination. PIs can learn great mentorship skills also from the NIH OITE series, but the corollary of this advice for PIs is that there is no place in a healthy lab environment for lab members who bully, harass, create discriminatory environments, or behave in unethical or unsafe ways. In short, you cannot easily mentor your way out of deeply toxic behavior and the longer such people are in an environment, the harder it can be to recover a safe and healthy lab culture.

Here, we provide 10 simple rules that we think are key to helping someone evaluate a lab environment and mentorship style that will let them thrive. For principal investigators (PIs), these 10 simple rules provide suggestions for how to align your lab culture and mentorship style to meet your vision for a lab mission and culture. Given the intensity of this evaluation, you and prospective mentors and labs will not have time to do this at the broad stage of first applying to programs/positions. Instead, this process is intended for when you are well into the process of acceptance to a graduate program (and/or have received offers for a post-bac or postdoctoral position). Each rule comes with several ways to evaluate the rule in a quick reference table (S1 Table), including preparation, what to look for during your evaluation, and example questions you can ask current or past lab members and PIs (with adjustment of questions for newly established PIs and labs) that get more detailed responses than something along the lines of “what are your expectations for graduate students?”. We focus here on the Ph.D. period, given it is the longest period of training, but hopefully these rules will be helpful to anyone considering a new environment, including a postdoctoral position or an industry position.

## Rule 1: Understand the mission of the lab

Although one can read papers that have been published by a lab and get an idea of what the lab has done, it can be hard to evaluate, without first-hand knowledge, the specific mission of the lab. The mission of the lab is the big-picture driving force that outlines what work the lab head will focus on when it comes to funding, project decisions, and collaborations. Some labs will be driven by a scientific question, whereas other labs will be driven by developing or using specific techniques. These lab differences will affect the training environment and your match for interests to it. For example, in a question-focused lab, people may have to adapt and learn new techniques and forge new collaborations to achieve their goal. In a technique-focused lab, people instead need to focus on the key and detailed aspects of a technology or technique and find interesting new applications or add novel advances. Some labs include training as an intrinsic part of the lab mission, which then influences the types of projects pursued. When considering a mission, consider how that aligns with your training goals for your career objective and how happy you will be pursuing and contributing to that mission – what it means both long term over the course of your training period, but also the daily and weekly aspects of the work that will keep you most excited to apply your hard work in that way.

## Rule 2: Understand the values of the lab

Where the mission of a lab is the rudder that sets the course of the ship, the values of the lab are the driving force – the way the ship gets to its destination. Although an organization, or people in an organization, can have a diverse set of values, there’s ultimately a few key values that truly matter that determine how the lab functions, makes decisions, and effects the daily and long-term outcomes for lab members. Example key lab values might be “innovation”, “impact”, “rigor”, “reproducibility”, “creativity”, or “collaboration”. Some of those values may speak more to you personally than other values. When you are in an organization whose key values are similar to your values, that is called values alignment. Research shows that organizations are much more effective and organization members are happier and more productive when their values are aligned with the organization [19]. Hence, a PI can create a more collegial and productive environment by assessing and creating a clear values structure of the lab, collaboratively with lab members. For scientists transitioning into a PI role, it can be beneficial to consider their key values as they design the short- and long-term goals of their research program. Additionally, it has been shown that even just sharing your value structure with another person can lead to a higher feeling of connectedness, even if values are not aligned [19]. So, at a minimum, sharing values amongst lab members can be a very helpful method for improving team dynamics. Evaluating the value structure of labs and departments early can help identify alignments that will help trainees thrive.

## Rule 3: Evaluate the psychological safety of a lab

A well-functioning lab has good communication, adapts as challenges arise, and handles potential conflict head-on. These traits exist when psychological safety is strong—where psychological safety at works means members feel safe to take risks and speak up without fear of repercussion. It has been found that the most innovative and productive teams share the trait of being psychologically safe [20]. Evaluating psychological safety may be the single best way to evaluate collegiality and innovation of a lab together with growth potential for you, since it means finding an environment of people who care about each other’s success and are willing to challenge you and offer candid feedback. In a psychologically safe environment, all lab members feel they can ask questions, challenge the status quo, take risks, and acknowledge mistakes without fear of harsh judgment. For example, someone might say “I forgot to order that reagent; I’ll improve my system” rather than hiding errors, because they have learned it is safe to admit mistakes. Psychological safety flourishes where leaders demonstrate humility, solicit feedback, maintain open communication, value contributions, and set clear expectations [19,20].

The goal here is to assess how safe people feel about speaking up, taking risks, and to characterize the response to challenging ideas, failure or mistakes, and the type of feedback delivered by the lab and the mentor. Some key indicators of psychological safety are seeing participation in way of questions and feedback from all lab members, but especially those with the least amount of power in a group structure. It is also important to evaluate the diversity of a lab along gender, nationality, religion, ethnicity, or other aspects of identity and assess whether psychological safety exists for all groups, or only preferentially one group [21]. For example, when you talk with men in the group you might find they have high levels of psychological safety, but women or gender nonbinary individuals may not share that same level of psychological safety.

## Rule 4: Evaluate the compatibility of the lab with your life- and work style

An advantage of the academic research lab, compared to typical industry positions, is there is typically a great deal of freedom of when and, sometimes where, you work. By their nature, some research projects may constrain work schedules, such as specific hours needed to complete a protocol or maintain animals. As a result, lab working culture can vary drastically. Some labs may have explicit working times when everyone is expected to be physically present, whereas other labs may have members that work almost entirely remotely or have sporadic in person working hours. In addition to working hours and location, lab culture also includes the timing and frequency of lab events, such as lab meetings and meetings with the mentor. What types of interactions and the expectation for how frequently or when these happen may be a poor match for what helps you thrive in work and outside of work. Mismatches in work style can significantly impact performance and well-being: structure-dependent trainees may struggle in flexible environments; newcomers benefit from overlapping hours with colleagues; night-owls underperform with mandatory early mornings; and poorly timed lab meetings can create work-life conflicts.

It is important to do some self-reflection about how you work and live best—what aspects of a schedule help you achieve your full potential in all aspects of your life? The goal here is not to eliminate labs from consideration that currently do not appear to match your perfect working conditions, but to evaluate whether you can thrive within a culture, e.g., what is the adaptability of a lab culture to shift and move to accommodate everyone within that lab to meet most of people’s needs most of the time? During this process, see if you can assess how the expectations are set, how it influences lab culture, and the flexibility of a culture to change around working hours and expectations. For PIs, this is one of the most important ways to use a lab expectations document [22]—setting clear expectations about working hours, availability by email or other communication system within and outside of typical hours, vacation policies, and how to communicate about adapting to individual and lab needs. PIs can further clarify commitment to lab environment by including the lab mission (Rule 1) and values (Rule 2) into this document.

## Rule 5: Understand decision-making with respect to projects, timelines, and collaborations

For as many diverse scientific questions that a lab can pursue, there are diverse approaches for deciding what projects to work on, how to work on them, how long to work on them for, and who to work on them with. In addition to those decisions, are also questions around how and when a trainee will apply for fellowships/awards, how/when to write manuscripts or present your research, and how/when to take on additional tasks like lab management or mentorship of another trainee. The mission and values of a lab will guide many of these decisions, but this rule is focused on helping you get to the nitty-gritty details that will help you understand and envision how your training period will unfold within a specific lab. Just as scientific missions and values vary by individual, your preferences for work style, project variety, and collaboration types are also highly personal and specific to you. An aspect of this rule is gathering data about yourself and the structure of a lab you are considering, to help you evaluate your confidence that decisions made will have your best interests in mind, along with satisfying the constraints that may exist around funding, lab mission, and feasibility. For training/career goals you might have—evaluate whether the mentor has previous experience helping people meet those goals and the decision tradeoffs around when those goals might conflict with larger goals and how that is navigated. For example, has the PI trained graduate students that publish strong first author papers in your field, win fellowships, and ultimately obtain jobs similar to those in which you are interested? For projects and collaborations – you want to understand how mentors and their trainees communicate and adjust when things become difficult or when it’s time for the next big step. For example, when and how is decided that it’s time to pivot when a technique or approach is consistently failing or when a collaboration becomes fraught with power dynamics, miscommunications, or divergence of opinions?

## Rule 6: Evaluate communication styles and adaptability

Communication is a key aspect of any training program—it’s important to understand your default communication style and that of a mentor and labmates. However, the goal here, like life and workstyle, is not to find a lab of assimilation (people who all approach communication the same way), but it is to assess the general awareness and adaptability that demonstrates that communication is healthy and will be a tool to navigate conflict and high stakes conversations. The NIH resilient scientist training noted above, offered for both trainees and mentors, covers a lot of ground on how to give and receive feedback and handle conflict. Much of this is built around specific ways and types of communication, adapting feedback styles for the type of conversation and for the person you are having it with. They offer these trainings broadly but also have them available on YouTube [18]. A good place for trainees and mentors to start is to do a self-evaluation of their default communication style along a grid. For example, using the OITE grid [23], do you tend to be direct and to the point (Driver) or do you need time to prepare for a meeting and think during the discussion (Analytical)? On a different dimension of style, do you ask a lot of questions and easily get off topic (Expressive) or do you soften feedback in unhelpful ways (Amiable). Once you have done some self-reflection, you can now look at a lab fit with respect to that communication style and evaluate whether there’s an awareness and adaptability to those styles to enable conflict resolution amongst and between people and to help provide and receive feedback in a productive way.

## Rule 7: Think about your present and your future

Sometimes, what works for us in the immediate future may look quite different than what is a good fit for us a year or several years from now. In graduate and postdoctoral training especially, we are on paths that are meant to stretch us and create deeply independent scholars. Hence, what may look appealing our first year in the lab and out of undergraduate training, lots of oversight and stepwise instructions for example, will rankle us in a few years after we’ve gained expertise through things like classes, research, conferences, and literature, which may go beyond our advisor’s experiences and expertise. Good mentors deliberately adjust their mentorship for mentee needs while maintaining high expectations, both between different mentees, and to the same mentee over time. It can be hard to picture where you will be in one to three years’ time, but asking targeted questions about how mentorship and experiences change over time can help you identify environments that will likely support your entire training period in the lab. For PIs, you can help assess the need for adapting mentorship more easily when you have clear and transparent communication. A starting point is the lab expectations document (Rule 4). Another method for helping establish this formally is collaborating with trainees on an annual Individual Development Plan (IDP) [24,25]. Depending on how you and your trainees use these resources, it is a great way to identify and adapt to changing needs.

## Rule 8: Thoroughly evaluate lab culture and mentorship from external sources

Lab cultures and mentors often have reputations that you hear about during interview visits or when talking with other members of the larger community. In fact, community members that are not directly in labs can have a highly informative view of a lab culture or mentorship style since they are farther removed from the power dynamics of the lab. However, it is important to assess this type of information thoroughly as it tends to be less specific and could represent a limited experience or even be inaccurate. Vague descriptions like “the fun lab” could mean collegial lunches and humor, or mandatory after-hours socializing with alcohol. Similarly, “high expectations” might indicate demanding availability and immediate responsiveness, or scientific rigor with structured mentorship toward excellence. These distinctions are critical for determining your potential fit in the environment.

Assuming you have asked a question and gotten a high-level (macroscopic) response, use that opportunity to drill down to understand how to interpret that statement so you can evaluate if it is relevant to you and if it matches the rest of the information you have gathered from the lab itself. Additionally, it can be important to try and separate fact from myth. Sometimes, macroscopic statements reflect something that has been passed down through the years and may not be particularly true, or if it was true, it’s no longer relevant. If you are hearing statements about a lab, see if you can get enough details about when and how that happened to understand it’s saliency, while being sensitive not to get into anything that would reveal personal details. You want to understand the general impressions of lab culture while avoiding the use of incorrect information when making your decisions.

## Rule 9: Weigh historical information accurately in your decision

A key factor about your decision to join a lab is to evaluate the experiences of current and past lab members. Just like every trainee is a unique individual, different trainees will have different experiences and thoughts about how happy and satisfied they are or were in a particular environment. Their experiences are important to understand, but more important to understand is how to assess someone else’s experiences as you attempt to predict what your experience in a lab will be. A particularly useful source of information about a lab and mentor are previous members of that lab. They have less of a conflict of interest and may have a longer-range perspective of the lab culture and mentoring experience.

Look for patterns in trainee experiences that might predict your own success: Do trainees typically complete their goals? Do mentors maintain positive relationships with alumni? Examine training duration, publication records, and award outcomes. A few outliers amid positive patterns suggest isolated issues, while consistent negative patterns indicate systemic problems. Mixed outcomes may signal the environment works well for specific trainee types—your task is determining which side of that divide you would fall on.

## Rule 10: Know the emergency exits

Despite careful evaluation, you may find yourself experiencing distress in your chosen lab—manifesting as avoidance behaviors, seeking external advice, excessive complaining, or declining mental health. While evaluating potential labs, identify the institutional support systems and policies (ombuds offices, department guidelines, human resources) that would facilitate transitioning between labs, if necessary. Additionally, measure the success of prior transitions and the culture around this—whether transitions have happened previously in the department and how they are viewed generally by people in the department. You may find some departments/institutions where transitions are not possible, some where they are possible but frowned upon, and others where it is done with care for all parties.

## Conclusion

These ten simple rules should help you efficiently evaluate potential research-mentor-lab combinations to find your optimal fit. The relative importance of each rule may vary depending on your career stage and trajectory. However, as minimum requirements you should identify labs and environments with psychological safety and free of discrimination. We hope that these rules, which were born out of our own experiences and the many experiences we have been entrusted with from colleagues, mentors, and mentees, can help trainees more quickly assess fit with the lab and the mentor, without having to go through the first-hand learning experiences themselves of discovering what does not work for them.

## Supporting information

S1 Table An Excel worksheet that contains columns for each rule that cover “Preparation/Homework”, “What to look for”, “Example questions for current trainees/alumni”, “Example questions for a PI”, “Example questions for a new PI”, and “Resources/Suggestions for PIs”. (XLSX)
